# Multi-site cardiac rhythm monitoring via multi-channel SCG system and exercise-induced physiological analysis

**DOI:** 10.3389/fphys.2026.1727160

**Published:** 2026-02-17

**Authors:** Miaoyang Hu, Huansheng Yi, Wenjun Yan, Wei Ren, Jianqi Wang, Fugui Qi

**Affiliations:** 1 Department of Cardiology, Xijing Hospital, Fourth Military Medical University, Xi’an, China; 2 Innovation Research Institute, Xijing Hospital, Fourth Military Medical University, Xi’an, China; 3 School of Biomedical Engineering, Fourth Military Medical University, Xi’an, China; 4 Hong Kong Generative AI Research and Development Center, Hong Kong University of Science and Technology, Hong Kong SAR, China; 5 Military Medical Innovation Center, Fourth Military Medical University, Xi’an, China

**Keywords:** cardiac cycle phases, cardiac rhythm monitoring, exercise-induced physiological, multi-channel, seismocardiogram

## Abstract

**Purpose:**

This study aims to achieve fine-grained multi-chamber monitoring and further investigate exercise-induced cardiac vibration patterns across thoracic sites through spatiotemporally resolved mechanical analysis and quantitative characterization, based on a custom-built multi-channel seismocardiography (MSCG) system integrated with electrocardiography (ECG). This approach holds clinical importance for early screening and dynamic management of cardiac structural abnormalities.

**Methods:**

1) For the multi-channel MSCG signals from continuous cardiac vibration monitoring across multiple thoracic regions (from our collected dataset and public dataset), a signal processing pipeline was established to extract temporal intervals and amplitude parameters from cardiac cycles. 2) These temporal disparities of corresponding feature points among channels, which could reflect the sequential mechanical activities of different cardiac chambers, were physiologically interpreted based on cardiac dynamic, thereby enabling a detailed analysis of chamber-specific timing relationships. 3) Furthermore, a pre- and post-exercise comparative protocol, analogous to stress ECG testing, was implemented to analyze multi-channel SCG feature changes and establish correlations between exercise-induced cardiac mechanical alterations and SCG parameters.

**Results:**

1) Synchronized multi-channel SCG and ECG signals were successfully captured by the custom-built high-precision dual-modal acquisition system, enabling precise identification of characteristic feature points across all channels. 2) Statistical analysis of cardiac cycles from individual subjects revealed that following exercise, humans exhibited significant forward shifts in aortic valve closure (AC) and mitral valve opening (MO) points across all five channels, consistent with exercise-induced heart rate elevation and shortened cardiac phases. 3) Regarding amplitude, the mitral valve site showed the earliest MC point, though no consistent spatial sequence emerged for other feature points during exercise.

**Conclusion:**

1) The spatiotemporal disparities across MSCG channels indeed correspond to the distinct physiological activities of the underlying cardiac chambers. 2) Exercise-induced cardiac mechanical dynamics were spatiotemporally resolved monitored by the system, revealing quantifiable timing shifts in valvular events undetectable by single-channel approaches. 3) The finding of consistent timing shifts in the AC and MO feature points under post-exercise scenario obtained via multi-channel monitoring, which supports the non-invasive assessment of cardiac function under exertion and in pathological conditions involving altered ventricular dynamics.

## 1 Introduction

Cardiovascular diseases (CVDs) remain the leading cause of global mortality (Ford et al., 2007), with an estimated mortality rate ranging from 230 to 1700 per 100,000 population, accounting for 24 ! to 47% of total deaths (Rosamond et al., 2007). There is significant research interest in chamber-specific monitoring of cardiac activities,which Including traditional electrocardiography (ECG) (Li et al., 2025) and video-based (Benezeth et al., 2024), and our proposed bio-radar (Zhang et al., 2019; Ren et al., 2025; Qiao et al., 2022). Extensive research has demonstrated that the mechanical vibration patterns of cardiac chambers and valves, as primary participants in cardiac physiological activities, provide spatially resolved and chamber-specific information crucial for cardiac health monitoring and disease diagnosis (Albu et al., 2024; Filgueiras-Rama and Jalife, 2016). Current clinical diagnostics primarily rely on ECG and ultrasound techniques. Ultrasound imaging provides chamber-specific visualization, unlike ECG which captures signals from a single point, but faces challenges such as high cost, lengthy procedures, or poor portability.

The evolution of cardiac mechanical monitoring began with the introduction of ballistocardiography (BCG) in 1961 (Starr and Wood, 1961), followed by Salerno et al.‘s development of seismocardiography (SCG) using sternum-mounted accelerometers (Salerno et al., 1991). Subsequent research efforts have focused on identifying characteristic waveform features corresponding to specific cardiac cycle phases through Doppler ultrasound validation (Marcus et al., 2007; Crow et al., 1994; Sørensen et al., 2011), including mitral valve opening (MO), aortic valve closure (AC), and rapid ejection (RE) phases. Later research of prevailing BCG/SCG-based methodologies predominantly employ single-point sternal accelerometry (Dubatovka and Buhmann, 2022), thereby capturing the superimposed signals from multiple cardiac chambers vibrations, and with efforts mainly focused on expanding the set of detectable characteristic points (Lin et al., 2018).

Spatially refined detection of multi-chamber cardiac vibrations can provide more fundamental and detailed information for sensing cardiac physiology and abnormalities. In contrast, the prevailing single-channel method (Işilay Zeybek et al., 2022; Dehkordi et al., 2019) cannot differentiate the origin chamber location of a detected abnormality, significantly limiting its utility for monitoring myocardial motion dynamics and deriving quantitative hemodynamic parameters. To overcome this limitation, Wen-Yen Lin et al. (Lin et al., 2018) pioneered a four-channel system positioned at clinical valve auscultation sites, but still mainly focused on discovering more new feature points based on the overall advantages of four channels. Recent advances have yielded multi-channel array-based MSCG systems, enabling multi-point surface vibration detection. Building upon these platforms, researchers have subsequently conducted temporal synchronization tests between MSCG and ECG (Munck et al., 2020; Sahoo et al., 2017), along with multi-dimensional visualization of temporal-spatial-directional vibration patterns across multiple thoracic locations through various analytical methods (Munck et al., 2019). Nevertheless, existing studies have mainly aimed to validate the system’s detection capability without anchoring their analysis in the vibratory patterns of cardiac chamber dynamics to interpret the temporal characteristics and the amplitude variations across multi-channel feature points, which is vital for understanding chamber-specific physiology and enabling precise cardiac monitoring.

More precisely, investigating the differential responses of cardiac chambers under exercise stress is of paramount importance for disease diagnosis, just as the Treadmill Exercise Test has become a well-established clinical cornerstone diagnostic paradigm (Kligfield and Lauer, 2006). By analyzing the timing of symptom onset, heart rate, and blood pressure response under controlled exercise loads, the Treadmill Exercise Test can reveal latent cardiac abnormalities that are undetectable at rest. For instance, exercise stress testing that evaluates electrocardiographic changes at target heart rates post-exercise can facilitate the diagnosis of coronary artery disease, assessment of cardiac function, and determination of disease severity (Guasch and Mont, 2016). However, ECG’s one-dimensional electrical signals cannot resolve chamber-specific mechanical alterations. A review of the literature confirms that no studies have yet investigated exercise-induced mechanical vibration changes via multi-channel SCG monitoring, nor have they provided a physiological interpretation of the resulting characteristic point variations.

To address these problems of multi-site cardiac rhythm monitoring and exercise-induced physiological analysis, the following series of work have been carried out: 1) A high-sensitivity and synchronized dual-mode system integrating multi-channel MSCG with ECG was developed, which successfully achieved continuous cardiac motion monitoring across multiple thoracic areas through synchronized multi-channel SCG acquisition, enabling precise identification of key cardiac cycle phases. 2) By establishing a signal processing pipeline to extract temporal intervals and amplitude parameters from SCG-derived cardiac cycles, and combining it with a physiological analysis of inter-channel temporal disparities during rest based on cardiac dynamics, this study validates the potential of multi-channel configurations for refined detection of chamber-specific structural characteristics. 3) This study presents a comparative analysis of multi-channel SCG features before and after exercise, establishing quantitative correlations between exercise-induced alterations in cardiac intervals/contractility and SCG parameters, thus facilitating physical exertion monitoring and prospective pathological assessment.

## 2 Materials and methods

### 2.1 System frameworks

**FIGURE 1:**
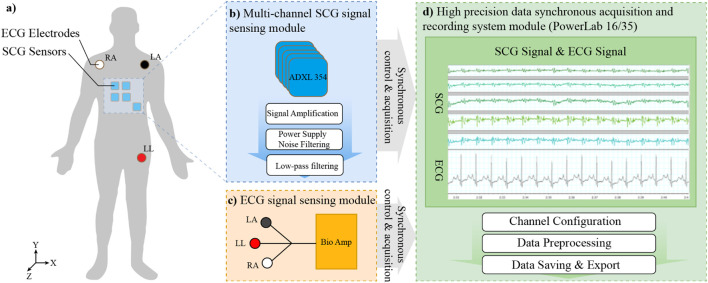
The schematic diagram of the electro-mechanical dual-modality detection system—— **(a)** The layout of all sensors and electrode. **(b)** The schematic diagram of multi-channel SCG signal sensing module. **(c)** The schematic diagram of ECG signal sensing module. **(d)** The schematic diagram of the high precision data synchronous acquisition and recording system module.

**FIGURE 2:**
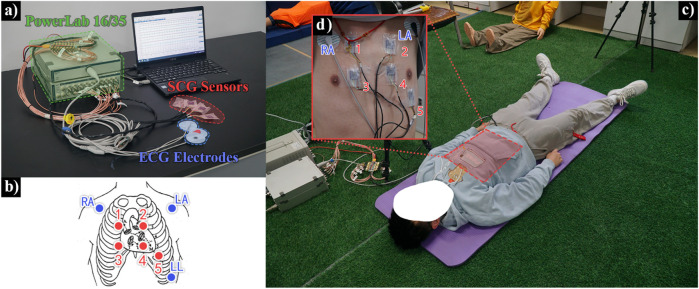
Experimental setup and acquisition equipment——**(a)** Schematic diagram of the acquisition system connection. **(b)** Schematic diagram of the relative positions of the SCG sensor module and ECG electrodes placed on the surface of the subject’s chest. **(c)** Experimental scene (supine position). **(d)** Actual placement effect of the SCG sensor module and ECG electrodes.

The electro-mechanical dual-modality monitoring system, as illustrated in Figure 1a, and the physical system is illustrated in Figure 2a, synchronously records subtle mechanical vibrations on the thoracic surface (resulting from cardiac and respiratory activities). This system, which consists of three key components: a multi-channel SCG signal acquisition module, a single-channel electrocardiography (ECG) acquisition module, and an integrated data acquisition module.

The multi-channel SCG signal sensing module consists of five accelerometers. As is demonstrated in Figure 1b, these sensors are placed at standard cardiac auscultation sites, including the mitral valve area, pulmonary valve area, aortic valve area, second aortic valve area, and tricuspid valve area. The ADXL355 accelerometer (from Analog Devices) is employed, which operates on a 2.25 V–3.6 V power supply and utilizes a built-in linear voltage regulator to provide a 1.8 V reference voltage for measurements. It outputs analog voltage values corresponding to X-, Y-, and Z-axis acceleration measurements. The sensors feature low noise density, minimal 0 g offset drift, high precision, and a ±2g measurement range, making them suitable for detecting weak thoracic vibrations. Their compact LLC package (6×6×2.2mm) and wide operating temperature range (−40 °C–125 °C) enable stable and secure placement on the thoracic surface. The sensors are electrically and mechanically isolated from one another to minimize interference from extraneous noise and power supply fluctuations.

The single-channel ECG signal acquisition module utilizes three patch electrodes to capture standard Lead II ECG signals. As is shown in Figure 1c, this module is capable of detecting bioelectric signals within a range of ± 5 µV to ± 100 µV. It is isolated from the power supply to ensure patient safety and integrates a low-noise, high-gain differential amplifier specifically optimized for biological signal measurement. Additionally, the module includes software-controlled low-pass, high-pass, and notch filters to reduce noise and useless frequency bands, enhancing signal quality for later processing and analysis.

The PowerLab 16/35 (From AD Instruments) module with exceptional precision, real-time performance, sampling rate, and synchronization was adopted, which could meet the high requirements of cardiac structural and physiological sensing (spatial multi-point, fine temporal resolution). As is shown in Figure 1d, which serves as the core unit for signal processing and recording. This device supports up to 16 input channels, with a total speed of 400,000 samples per second and 16-bit resolution. It communicates with a host computer via High Speed USB (2.0) and offers both BNC-standard single-ended inputs and four differential input interfaces for enhanced compatibility. The system also includes dual independent analog output channels capable of generating predefined waveforms such as square pulses, triangular waves, sine waves, and step waves, as well as user-defined custom waveforms through software configuration. Designed for high-fidelity signal reproduction, this setup ensures precise and synchronized acquisition of mechanical and electrical cardiopulmonary signals, enabling comprehensive physiological monitoring and analysis.

### 2.2 Data preprocessing

The original data contains interference noise caused by environmental factors such as ambient noise and bioelectricity signals. To obtain relatively high-quality SCG and ECG signals, it is necessary to filter these noise sources. Specifically:

- SCG Signal Processing: This system employs a Butterworth band-pass filter with cutoff frequencies of 0.9 Hz–90 Hz, applied uniformly across all SCG signals to remove out-of-range noise.
- ECG Signal Processing: While hardware preprocessing had already applied filtering and amplification for ECG signals, minimal subsequent processing was required. A simple high-pass filter (60 Hz) sufficed to mitigate breath-like interference, yielding clean ECG signal outputs.

Additionally, perform downsampling on each dataset to improve the efficiency of subsequent data analysis. Organize and store each dataset in the corresponding format file for further analysis using MATLAB.

The signal is segmented based on the ECG R-peak markers. Using each R-peak as a reference point, a time window extending 100 m before and 500 m after the R-peak is extracted from the entire signal, resulting in individual segments each lasting 0.6 s. After completing the segmentation, these segments are concatenated into an m×n matrix, where n is the length of each individual segment and m is the total number of segments.

### 2.3 Searching for SCG feature points

When discussing the composition of SCG signals, it can be observed that they are primarily composed of two wave groups, S1 and S2. The formation of the S1 wave group is related to the contraction process of the heart, while the S2 wave group is associated with the relaxation process of the heart. Within these two wave groups, the minor vibrations caused by the opening and closing of the heart valves and blood flow are factors that cannot be ignored. These vibrations are transmitted through muscles and organs to the surface of the chest cavity and manifest on the SCG image as a series of continuous peaks and troughs of acceleration, with each peak and trough potentially indicating specific cardiac physiological events.

This study focuses on four key feature points in the SCG signal: Aortic valve opening (AO), Aortic valve closure (AC), Mitral valve opening (MO), and Mitral valve closure (MC). These points significantly reflect the heart’s physiological activities. To precisely identify these feature points, We adopted and improved the feature point localization algorithm from reference (Rienzo et al., 2017) to locate each channel of the SCG signal. The specific algorithmic procedure is described as follows.

**FIGURE 3:**
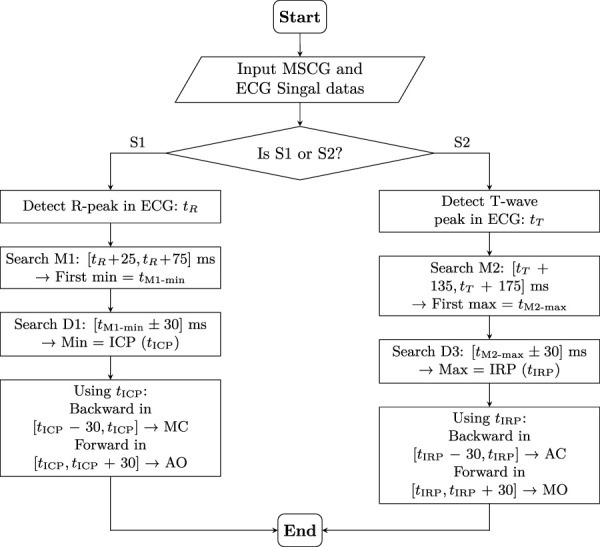
Feature point extraction process in SCG signal based on ECG fiducial points. S1-related events (MC, AO) and S2-related events (AC, MO) are detected using a two-stage search strategy anchored to R-peak $\left(t_{R}\right)$ and T-wave peak $\left(t_{T}\right)$ , respectively.

As showing in Figure 3 for the S1-related feature detection, the R-peak in the ECG signal is first detected, and its occurrence time is denoted as $t_{R}$ . This time point serves as the initial temporal reference for all SCG channels. A search window $\text{M}1=\left[t_{R}+25\text{ ms},\;t_{R}+75\text{ ms}\right]$ is applied to each SCG channel to locate the first minimum value within this interval. This minimum point is then designated as a refined reference, denoted as $t_{\text{M}1-\text{min}}$ . Subsequently, a symmetric search window $\text{D}1=\left[t_{\text{M}1-\text{min}}-30\text{ ms},\;t_{\text{M}1-\text{min}}+30\text{ ms}\right]$ is centered on $t_{\text{M}1-\text{min}}$ , and the minimum value within D1 is identified and labeled as the isovolumic contraction point (ICP), with its time denoted as $t_{\text{ICP}}$ . Using $t_{\text{ICP}}$ as the new anchor, two additional searches are conducted:

- A forward search in the interval $\text{D}2=\left[t_{\text{ICP}},\;t_{\text{ICP}}+30\text{ ms}\right]$ is performed to find the first peak (maximum value), which is marked as the AO (aortic valve opening) point.
- A backward search in the interval $\text{D}3=\left[t_{\text{ICP}}-30\text{ ms},\;t_{\text{ICP}}\right]$ is used to detect the first peak (maximum value) preceding $t_{\text{ICP}}$ , which is identified as the MC (mitral valve closure) point.

For the S2-related feature detection, the peak of the T-wave in the ECG signal is detected, and its occurrence time is denoted as $t_{T}$ . This time point serves as the initial reference for the S2 analysis. A search window $\text{M}2=\left[t_{T}+135\text{ ms},\;t_{T}+175\text{ ms}\right]$ is applied to each SCG channel to locate the first maximum value within this interval. This maximum point is denoted as $t_{\text{M}2-\text{max}}$ . A new search window $\text{D}3=\left[t_{\text{M}2-\text{max}}-30\text{ ms},\;t_{\text{M}2-\text{max}}+30\text{ ms}\right]$ is then centered on $t_{\text{M}2-\text{max}}$ , and the maximum value within this window is selected and labeled as the isovolumic relaxation point (IRP), with its time denoted as $t_{\text{IRP}}$ . Using $t_{\text{IRP}}$ as the reference:

- A forward search in the interval $\text{D}4=\left[t_{\text{IRP}},\;t_{\text{IRP}}+30\text{ ms}\right]$ identifies the first trough (minimum value), designated as the MO (mitral valve opening) point.
- A backward search in the interval $\text{D}5=\left[t_{\text{IRP}}-30\text{ ms},\;t_{\text{IRP}}\right]$ identifies the first peak (maximum value) before $t_{\text{IRP}}$ , which is marked as the AC (aortic valve closure) point.

## 3 Experimental setup

To investigate the spatiotemporal patterns of MSCG signals across multiple channels under various respiratory modes, we designed and implemented the following experimental protocol with rigorous methodological considerations.

### 3.1 Pre-experimental preparations

Participants were instructed to abstain from vigorous physical activities that might induce significant heart rate fluctuations for at least 1 hour prior to the experiment, maintaining their heart rate within natural physiological ranges. As illustrated in Figures 2b,d, five SCG sensing modules were precisely positioned over the thoracic surface corresponding to five cardiac auscultation areas: the mitral valve area, pulmonary valve area, aortic valve area, second aortic valve area, and tricuspid valve area. The sensing modules were securely affixed to ensure complete adhesion to the thoracic surface. Concurrently, three disposable ECG electrodes were placed on the left arm (LA), right arm (RA), and left lower limb (LL) following standard Lead II configuration, which direct connected to the data acquisition system.

### 3.2 Experimental procedure

**FIGURE 4:**
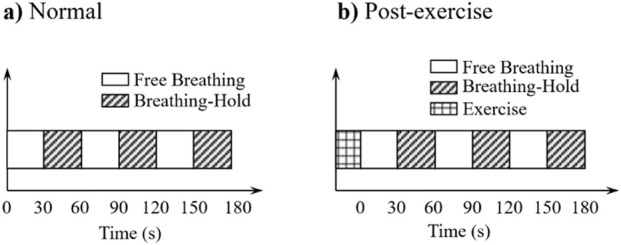
The two different scenarios timing diagram. ——**(a)**The timing diagram in normal scenario. **(b)**The timing diagram in post-exercise scenario.

When a set of experimental data acquisition commences,the subject is required to assume a supine posture (as shown in Figure 2c) for data acquisition process, and keep this posture unchanged during subsequent scenarios. The experiment will be conducted in two different scenarios: normal scenario and post-exercise scenarios. The subject’s SCG and ECG signals are synchronously recorded using the experimental equipment. The specifications for each scenario are as follows:

- Normal Scenarios: Participants remain in the supine position throughout the entire experiment and maintain a quasi-static state. Before the experiment begins, participants are asked to adjust their breathing rate to a relatively natural rhythm and sustain it until data collection is complete. When data acquisition starts, participants first breathe normally for 30 s, followed by 30 s of breath-holding. This process is repeated. As shown in Figure 4a, three times in total, concluding after 3 min of data collection.
- Post-exercise Scenario: Participants start with performing 10 push-ups prior to the experiment. Immediately following the exercise, they adjust their body position to the supine posture and maintain a quasi-static state until data collection end. At the beginning of data acquisition, participants first breathe at their current respiratory rate for 30 s, then hold their breath for another 30 s. This cycle, as shown in Figure 4b, is repeated three times in total, concluding after 3 min of uninterrupted data collection.

For each complete experimental session, data acquisition is initially conducted under the Normal Scenario. Following a standardized 10-min recovery interval, the subject subsequently undergoes data acquisition under the Post-exercise Scenario, thereby constituting a full cycle of the experimental protocol.

### 3.3 Baseline characteristics of subjects

The experiment included three healthy male subjects (coded SUB01 to SUB03) with corresponding information shown in Table 1. Resulting in a total of 431 cardiac cycle samples (Table 2). The mean age of all the participants is approximately 25 years old and none of them had any history of pulmonary or cardiovascular diseases. This study was performed in line with the principles of the Declaration of Helsinki. The Ethic Committee of the First Affiliated Hospital of the Fourth Military Medical University approved the study (No. KY20253449-1). The informed consent of all subjects was obtained prior to their participation.

**TABLE 1:** Baseline characteristics of subjects.

ID	Sex	Age	Ht	Wt	BMI	Smoking^a^	Disease^b^
SUB01	Male	33	178	75	24.2	No	None
SUB02	Male	27	180	72	22.2	No	None
SUB03	Male	24	175	68	21.0	No	None

^a^ History of smoking.

^b^ History of or current cardiopulmonary disease.

**TABLE 2:** The number of cardiac cycle samples.

ID	Normal^a^	Post exercise^b^	Total samples
SUB01	64	68	132
SUB02	82	67	149
SUB03	88	62	150

^a^ The number of cardiac cycle samples on each channel (ECG,SCG-Ch1 SCG-Ch5) for all the experimental groups in the normal scenario of current subject.

^b^ The number of cardiac cycle samples on each channel for all the experimental groups in the post exercise scenario of current subject.

**FIGURE 5:**
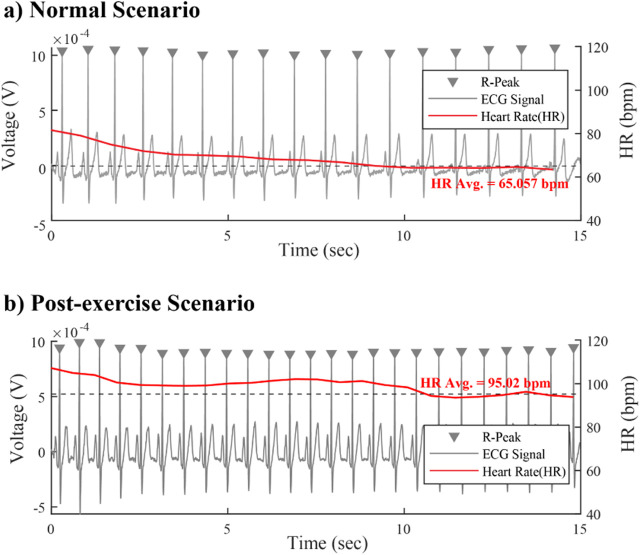
The baseline heart rate of subjects——**(a)**The trend and average heart rate in Normal scenario. **(b)**The trend and average heart rate in Post-exercise scenario.

The baseline heart rate of one representative participant under the two experimental scenarios show in Figures 5a,b respectively. To ensure a clear physiological distinction between pre- and post-exercise states, we instructed the participant to perform consecutive push-ups until their heart rate increased to approximately 1.5 times their resting baseline and remained stable at that level for a sustained period.

### 3.4 Validation on public datasets

**FIGURE 6:**
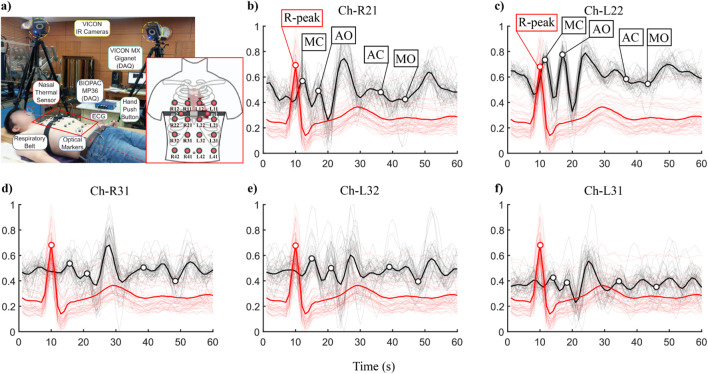
Experimental Setup and Data Preview of Public Dataset——**(a)** Experimental Setup. **(b–f)** the ECG and SCG signal segments overlapped plots of each channel.

To further validate our proposed method, we introduced an public dataset for verification. The dataset we used is from (Shafiq and Chakravarthy Veluvolu, 2017), in which chest vibration signals were collected from 16 different points on the human thoracic surface using a VICON motion capture system. Synchronously, nasal breathing and ECG signals were recorded. Their experimental setup is shown in Figure 6a. This dataset contains 72 trials from 11 male participants, with a cumulative duration of approximately 215 min under various conditions such as normal breathing, breath-holding, irregular breathing, and post-exercise recovery. We selected five subjects’ data of the dataset, which recorded both pre- and post-exercise breathing scenarios and their subject ID and personal information is provided in Table 3.

**TABLE 3:** Baseline characteristics of subjects of the public dataset.

ID	Sex	Age	Ht	Wt	BMI	Smoking^a^
S3	Male	26	168	63	22.3	Yes
S4	Male	22	178	65	20.5	Yes
S6	Male	22	168	61	21.6	No
S7	Male	32	170	53	18.3	No
S8	Male	23	158	68	27.2	No

^a^ History of smoking.

For these data, only the Z-axis signals from channels R21, R31, L22, L32 and L31 were utilized. Compared with our collected dataset, the public dataset contains considerable interference in the signals due to its acquisition via a non-contact optical method, including noise and baseline drift. Therefore, additional processing was required. We filtered the raw data using a 4th-order low-pass Butterworth filter with a cutoff frequency of 20 Hz and removed baseline drift with MATLAB’s built-in detrend function. Subsequently, we performed a second-order differentiation operation on all the displacement signals to convert them into the final SCG signals. The signals were segmented according to R-peak, normalized and visualized in the form of overlapped plots. The red bold lines and the black bold lines respectively represent the mean values of the ECG and SCG signal segments (Figures 6b–f, using subject S3 as an example).

## 4 Results

### 4.1 Identification of multi-channel SCG feature points

**FIGURE 7:**
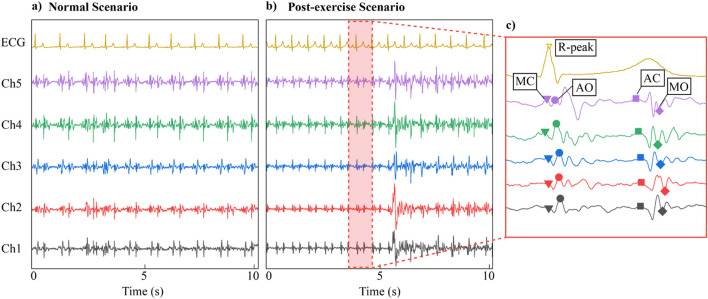
Data preview of the experiment——**(a)** Normal scenario multi-channel SCG and ECG raw data. **(b)** Post-exercise scenario multi-channel SCG and ECG raw data. **(c)** Search results of key points MC, AO, MO, AC on SCG signals.

Figure 7 displays the signals collected from each channel during the experiment. Figure 7a presents 10-s data under normal breathing conditions, while Figure 7b shows the data for the post-exercise scenario. The results reveal clear and rhythmic waveforms across all channels, with the SCG signals (Ch1-Ch5) exhibiting high correlation with the ECG reference. Furthermore, distinct morphological differences are evident between the two scenarios. Figure 7c shows the result of zoom in one of the cardiac cycles of the signal. For the SCG signal, four significant feature points are identifiable in the SCG signal within a cycle: MC, AO, AC, and MO, which correspond to key physiological events in the cardiac activity cycle, namely, mitral valve closure, aortic valve opening, aortic valve closure, and mitral valve opening, respectively.

### 4.2 Changes in SCG feature parameters before and after exercise

To investigate exercise-induced changes in SCG characteristic parameters, an observational analysis was conducted on three subjects under both normal and post-exercise scenario.

**FIGURE 8:**
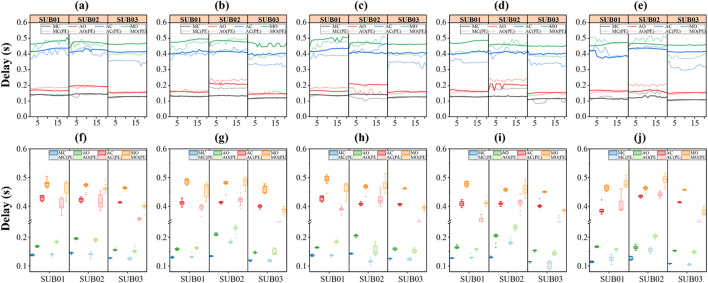
Comparison of time delays for different targets across multiple channels——**(a–e)** is the time delays of MC, AO, AC and MO feature points from R peak in each channel (Ch1-Ch5) obtained by processing. **(f–j)** are the corresponding box-type statistical plots.

The average time differences between each feature point and the R peak in both scenarios are summarized in Tables 4, 5. Time delays of feature points (MC, AO, AC, MO) relative to the R peak across all channels are visualized in Figure 8. The results indicate that, for all subjects and across all five channels, the AC and MO points shifted earlier after exercise. This aligns with normal physiological responses: increased heart rate post-exercise shortens systolic and diastolic periods, leading to faster ventricular pressure decline, earlier aortic valve closure (AC), and earlier mitral valve opening (MO).

**TABLE 4:** Average time difference between each feature point and the R peak under normal and post-exercise scenarios.

Subject ID	Channels	Normal				Post exercise			
		MC	AO	AC	MO	MC	AO	AC	MO
SUB01	Ch1	0.14019	0.16957	0.41478	0.46101	0.14985	0.20431	0.38598	0.43689
SUB01	Ch2	0.13057	0.17206	0.39762	0.46541	0.13504	0.17567	0.37742	0.44074
SUB01	Ch3	0.13856	0.16560	0.41703	0.47370	0.14858	0.20502	0.37171	0.43588
SUB01	Ch4	0.12850	0.16934	0.39830	0.45834	0.13285	0.17658	0.36188	0.42312
SUB01	Ch5	0.11251	0.17083	0.41600	0.47021	0.14298	0.18970	0.39205	0.44978
SUB02	Ch1	0.13372	0.16293	0.46611	0.53695	0.12791	0.17685	0.36958	0.42274
SUB02	Ch2	0.12778	0.15426	0.43571	0.51093	0.12615	0.16630	0.32990	0.39647
SUB02	Ch3	0.12739	0.15834	0.43266	0.48409	0.12364	0.16327	0.35584	0.41638
SUB02	Ch4	0.12046	0.14799	0.46281	0.51699	0.12030	0.15542	0.34964	0.40760
SUB02	Ch5	0.10797	0.13795	0.45609	0.52333	0.10651	0.14163	0.31350	0.36552
SUB03	Ch1	0.12879	0.15665	0.41513	0.46647	0.12666	0.15217	0.35257	0.40646
SUB03	Ch2	0.12089	0.14708	0.42452	0.47242	0.11859	0.14429	0.34665	0.40337
SUB03	Ch3	0.12669	0.16091	0.40737	0.46294	0.12428	0.15499	0.35003	0.40947
SUB03	Ch4	0.11520	0.15501	0.41755	0.47693	0.11007	0.14730	0.35202	0.40286
SUB03	Ch5	0.10902	0.15795	0.41530	0.45677	0.10683	0.15075	0.33704	0.40545

**TABLE 5:** Average time difference between each feature point and the R peak under normal and post-exercise scenarios of the public dataset.

Subject ID	Channels	Normal				Post exercise			
		MC	AO	AC	MO	MC	AO	AC	MO
S3	Ch1	0.07898	0.14293	0.33602	0.39890	0.11242	0.16164	0.37668	0.43749
S3	Ch2	0.08041	0.14385	0.38823	0.46905	0.10530	0.16125	0.37579	0.42984
S3	Ch3	0.11692	0.16680	0.42420	0.50051	0.14562	0.19810	0.32649	0.38556
S3	Ch4	0.11203	0.16415	0.42979	0.49571	0.14336	0.19756	0.34042	0.40117
S3	Ch5	0.09996	0.15902	0.42306	0.47874	0.12652	0.17133	0.37878	0.44571
S4	Ch1	0.12191	0.16342	0.35346	0.41241	0.12182	0.18096	0.36712	0.41955
S4	Ch2	0.10060	0.17112	0.43362	0.49931	0.12860	0.23151	0.36208	0.42153
S4	Ch3	0.12658	0.16667	0.37382	0.43749	0.15502	0.24092	0.30985	0.42403
S4	Ch4	0.12655	0.17648	0.37235	0.44527	0.15327	0.24453	0.42352	0.48099
S4	Ch5	0.11569	0.17388	0.37670	0.44108	0.14720	0.22791	0.42760	0.49514
S6	Ch1	0.07492	0.13654	0.38600	0.45798	0.08205	0.18146	0.35116	0.41946
S6	Ch2	0.06064	0.13038	0.36250	0.44480	0.12760	0.20012	0.43162	0.49753
S6	Ch3	0.10336	0.15396	0.38607	0.47047	0.12211	0.18751	0.38007	0.45384
S6	Ch4	0.09742	0.15226	0.37086	0.45751	0.11677	0.17235	0.35521	0.42865
S6	Ch5	0.05955	0.13196	0.34294	0.45024	0.13900	0.20912	0.32568	0.41417
S7	Ch1	0.06287	0.11910	0.43737	0.50877	0.16485	0.21587	0.33054	0.40693
S7	Ch2	0.12653	0.17194	0.43957	0.50987	0.17078	0.22353	0.33094	0.39942
S7	Ch3	0.09474	0.15078	0.39172	0.45275	0.10669	0.16167	0.25913	0.32188
S7	Ch4	0.08285	0.15016	0.46373	0.53865	0.08920	0.13754	0.23916	0.30630
S7	Ch5	0.07474	0.13705	0.45228	0.52596	0.16171	0.21714	0.23102	0.30114
S8	Ch1	0.13310	0.18893	0.41987	0.49002	0.10050	0.17063	0.35336	0.42290
S8	Ch2	0.09839	0.14301	0.41270	0.48115	0.10548	0.16875	0.34106	0.40775
S8	Ch3	0.14642	0.20291	0.44159	0.52220	0.17995	0.23951	0.30457	0.37688
S8	Ch4	0.12534	0.16810	0.42525	0.51025	0.17250	0.25269	0.38804	0.45626
S8	Ch5	0.11348	0.15607	0.41952	0.49397	0.13140	0.22635	0.35423	0.43325

**FIGURE 9:**
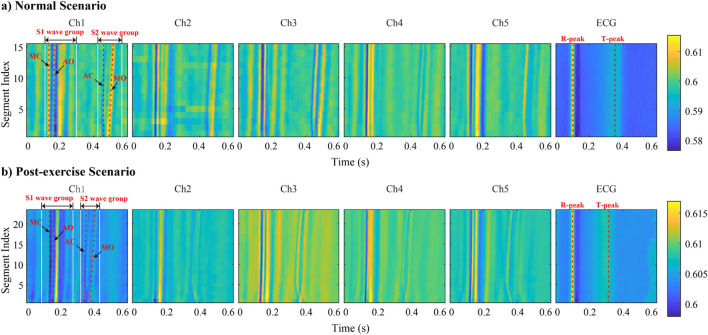
Data preview of the experiment within heatmap of cardiac cycle segments——**(a)** Normal scenario multi-channel SCG and ECG heatmap. **(b)** Post-exercise scenario multi-channel SCG and ECG heatmap.

To enable refined observation and comparison of the correspondence between MSCG-derived cardiac cycle characteristic points and ECG references, a series of cardiac cycle segments are visualized as heatmaps in Figure 9. The previously collected and segmented data are arranged by channels (Ch1 Ch5 and ECG). Datas are grouped into Normal Scenario and Post-exercise Scenario, with each group undergoing normalization to compare the energy distribution differences across channels. In the figure, the horizontal axis represents time, the vertical axis represents the segment number of the signal data, and the color intensity indicates the strength of the signal energy at that time point. By comparing the energy distributions across different channels in the figure, for the ECG channel, we can observe the clear appearance of R peaks and T peaks. Moreover, under the post-exercise scenario, the distance between the ECG R peak and T peak is closer compared to the normal scenario. For MSCG channels (Ch1 Ch5), the presence of wave groups S1 and S2 can be clearly observed, and compared to the Normal Scenario, under the Post-exercise Scenario, S2 noticeably moves closer to S1. Additionally, over time, the interval between S1 and S2 gradually increases, which aligns with the aforementioned trend of changes.

## 5 Discussion

In this study, several key innovations in the field of cardiac monitoring are presented: A highly sensitive multi-channel MSCG system was constructed, successfully obtaining synchronous multi-channel SCG and achieving continuous multi-channel cardiac motion monitoring. Secondly, this study indicated that the SCG based on MSCG can precisely determine the critical stages of the cardiac cycle, the cardiac time intervals extracted from the cardiac cycle based on SCG can reflect the alterations in cardiac time intervals, indicating its potential in cardiac rhythm and motion monitoring and its possible role in cardiac disease surveillance. Thirdly, multi-channel SCG was collected based on the valve auscultation areas, and certain patterns were observed, as well as some issues. This can guide further investigations on the valve collection points suitable for the characteristics of SCG to accurately detect the physiological activities of different valves.

### 5.1 Exercise-induced temporal shifts in SCG feature points

The SCG signals collected from each channel could accurately identify four key feature points: AO, AC, MO, and MC, thereby reflecting cardiac physiological activities. Theoretically, it can determine the key stages of the cardiac cycle and be employed to assess cardiac time intervals and functional indicators such as isovolumic contraction time (IVCT), left ventricular ejection time (LVET), and isovolumic relaxation time (IVRT) (Taebi et al., 2019), but further verification with synchronous cardiac ultrasound is required. We performed a comparative analysis of SCG feature points under normal and post-exercise scenario. The results indicated a consistent forward shift in AC and MO points across all five channels and all three subjects after exercise. This aligns with normal physiological responses: increased heart rate shortens both systolic and diastolic durations, leading to a more rapid decline in ventricular pressure and consequently earlier valve events. Moreover, these mechanical timing shifts mirror changes observed in certain pathological conditions, such as mitral regurgitation or ventricular septal defects, wherein abnormal blood flow also causes premature valve closure or opening. This supports the potential utility of our MSCG system in non-invasive assessment of cardiac function during stress and its application in detecting pathological ventricular pressure dynamics.

### 5.2 Spatial variability and interpretation challenges in multi-channel SCG signals

Previous studies used a single accelerometer on sternum, in conjunction with ECG, phonocardiogram, or echocardiogram, for signal acquisition (Wick et al., 2011; Wick et al., 2012). This study also explored spatiotemporal differences in SCG signals across multiple channels. Compared with previous studies in which signals of a single spot were acquired, multichannel SCG can record more details of each part of a heart, such as differential feature point signals of each particular sites and their time delay. Moreover, we detect more location-specific motion of each valve or chamber instead of picking up the composite vibration signals of a whole heart in the conventional sternal SCG. We observed that the mitral valve site (Ch5) consistently exhibited the earliest MC point during rest. However, no uniform spatial pattern was identified for other feature points or under exercise conditions. The variability in timing of the same feature point across channels suggests that SCG signals are influenced by multiple factors, including the physical distance between sensors and vibration sources, anatomical differences, and the composite nature of thoracic vibrations—which integrate valvular, myocardial, and blood flow components. Importantly, traditional auscultation sites are optimized for acoustic rather than mechanical detection, and may not ideally align with the optimal locations for vibration sensing. Therefore, while multi-channel SCG offers richer spatial information than single-point methods, further research is needed to determine optimal sensor placements specifically for SCG, and to correlate mechanical events with anatomical structures using modalities such as echocardiography.

## 6 Conclusion

The spatiotemporal disparities across MSCG channels indeed correspond to the distinct physiological activities of the underlying cardiac chambers. Exercise-induced cardiac mechanical dynamics were spatiotemporally resolved monitored by the system, revealing quantifiable timing shifts in valvular events undetectable by single-channel approaches. The finding of consistent timing shifts in the AC and MO feature points under post-exercise scenario obtained via multi-channel monitoring, which supports the non-invasive assessment of cardiac function under exertion and in pathological conditions involving altered ventricular dynamics.

## Data Availability

The original contributions presented in the study are included in the article/supplementary material, further inquiries can be directed to the corresponding author.
